# Finding “What's Wrong With Us”: Antibiotic Prescribing Practice Among Physicians in the United States

**DOI:** 10.3389/fsoc.2020.00005

**Published:** 2020-02-18

**Authors:** Katharina Rynkiewich

**Affiliations:** Department of Anthropology, Washington University in St. Louis, St. Louis, MO, United States

**Keywords:** antibiotic stewardship, antibiotic prescribing, social theory, antimicrobial resistance, social determinants, physician behavior change

## Abstract

Antibiotic stewardship—or the responsible use of antibiotics—has been touted as a solution to the problem of antibiotic resistance. Antibiotic stewardship in medical institutions attempts to change the antibiotic prescribing “behaviors” and “habits” of physicians. Interventions abound targeting “problem prescribers,” or those physicians whose practice is out of line with physician peers. Thus, the locus of decision-making in antibiotic prescribing is thought to be the found with the individual physician. Based on 18 months of participant observation and in-depth interviewing of antibiotic-prescribing physicians at two medical institutions in the United States, this paper will question notions of antibiotic stewardship that center on individual “behaviors” and “habits.” Many physicians have taken to heart a reductionist approach in studies of antibiotic prescribing, including several physicians I encountered during research who enthusiastically located the benefit of my research in the ability to identify “what's wrong with us.” In this paper, I use two representative ethnographic case studies to argue that antibiotic stewardship interventions aimed at identifying and correcting “bad” physician practice limit the possibilities of understanding the social dynamics of the institution. Through an analysis of everyday encounters in the hospital setting, I show how decision-making in antibiotic prescribing can more productively be located between and among institutions, physicians, patient charts, and other hospital-based staff members (e.g., pharmacists, nurses). By demonstrating that *antibiotic prescribing is a collective practice* occurring through engagement with social and material surroundings, I argue that we can better account for the weighted ways in which social action and relations unfold over time.

## Introduction

“*Antibiotic stewardship is very complex. Half of it is psychology. How do you make people do what they don't want to do? This is not medicine, it's not evidence-based medicine which is the thrust of what we were trained to do. I feel sometimes like a salesperson figuring out how people think. I don't understand this. This is a completely different field.” -Infectious disease practitioner*

Antibiotic resistance is a global threat to our health and well-being. Though resistance to antibiotics is not a new phenomenon, only recently have countries like the United States begun to take on antibiotic oversight as one of the defining issues of our time. In recent approaches to combating antibiotic resistance in the United States, there has been a central focus on the policy of antimicrobial or antibiotic stewardship—the responsible use of antibiotics. Antibiotics are overused and misused on a regular basis, and thus antibiotic stewardship endeavors to bring errant use of antibiotics into line with appropriate practice.

In attempts to correct inappropriate practice, antibiotic stewardship teams^1^ in medical institutions use interventions to target the antibiotic prescribing “behaviors” and “habits” of physicians. For example, if a physician overuses ceftriaxone by prescribing every patient to take 10 days of the antibiotic, the antibiotic stewardship team might utilize careful messaging to get the physician to switch how they prescribe ceftriaxone. Sometimes, antibiotic stewardship can intervene in simple ways that reduce overall antibiotic use. However, as this paper illustrates, there are shortcomings with this approach to antibiotic optimization. Primarily, antibiotic stewardship that identifies the crux of the problem with antibiotic prescribing as originating in the individual physician (i.e., their thoughts and behaviors) leans on a fallacy: though a single physician may sign a prescription order for antibiotics, they are likely not the only person considering, discussing, and ultimately deciding on antibiotic therapy for the patient.

Through the use of ethnographic data collected during fieldwork at two medical institutions in an urban midwestern setting in the United States, I will demonstrate how individual physicians operate within a complex web of relationships and institutional protocols that emphasize the distributed, collective nature of antibiotic prescribing. I will use two representative ethnographic case studies to show that *antibiotic prescribing is a collective practice* occurring through engagement with social and material surroundings.

Social science research has established that there is a myriad of factors, such as professional influence (Livorsi et al., 2015; Papoutsi et al., 2017) and communication styles (Linkin et al., 2007; Skodvin et al., 2017), that go into antibiotic decision making in medical settings. Drawing on this research and the data presented in this article, I propose that antibiotic stewardship interventions could be improved through greater acknowledgment and integration of the social dynamics of the institution. Thus, I argue that antibiotic stewardship interventions aimed at identifying and correcting “bad” physician practice limit the possibilities of understanding the ways in which physicians are interconnected and interdependent in their practices of antibiotic prescription.

## The Foundations of Antibiotic Stewardship

Antibiotic agents have been in circulation since the advent of sulfonamide drugs in the early twentieth century (Barrett and Armelagos, 2013; Podolsky, 2015). Antibiotics have minimized the threat of infectious diseases while they simultaneously encourage antibiotic resistance. Overuse and misuse of antibiotics has led to what is commonly referred to as a “crisis” of antibiotic resistance (Neu, 1992; Ventola, 2015; Mendelson et al., 2017). Physicians and researchers have long cited the dangers of antibiotic use (Hardin, 1968; see Barrett et al., 1998). However, little oversight of antibiotic use has been achieved globally. In fact, there are only two major antibiotic oversight programs worth mentioning: antibiotic control programs and antibiotic stewardship.

Antibiotic control programs in the United States began in the 1970's (Haley et al., 1985; Podolsky, 2015) and involved measuring institutional use of antibiotics. The control programs largely aimed to alter institutional use of antibiotics by regulating access and purchasing. Control programs in the United States were critiqued for their inability to enforce change at the institutional level due to the powerful resistance of the pharmaceutical industry (cf. Podolsky, 2015). Eventually, antibiotic control programs were integrated into infectious disease divisions with specialized pharmacy staff. Antibiotic stewardship, introduced in the mid-1990's, was intended as an expansion of influence over antibiotic use for specialists such as infectious disease physicians and pharmacists.

Antibiotic stewardship first appeared in the medical literature in McGowan and Gerding (1996), where it was described as “the limitation of use of inappropriate agents, but also the proper use, dosing, and duration of antimicrobial agents to achieve optimal efficacy in treating and preventing infections” (p. 371). Early definitions of antibiotic stewardship highlighted the potentially global impact of reducing antibiotic use. The association between the use of antibiotics and the emergence of antibiotic resistance has since catapulted antibiotic stewardship into a standard in medical practice. In the past 20 years, antibiotic stewardship has been heavily endorsed by international organizations and governments (Mendelson et al., 2017).

In the United States, a government-issued report detailing plans to combat antibiotic resistance was published in 2015. The Centers for Disease Control and Prevention (CDC) has published several guidelines for antibiotic stewardship in medical institutions including hospitals (Centers for Disease Control Prevention, 2014), nursing homes (Centers for Disease Control Prevention, 2015), and outpatient settings (Centers for Disease Control Prevention, 2016b). Additionally, the CDC has created an online education program for antibiotic stewardship (Centers for Disease Control Prevention, 2016a). There is now a medical management standard for the policy meaning that institutions accredited by The Joint Commission must maintain an antibiotic stewardship team that follows established guidelines as set by the accreditation agency.

### What Is Antibiotic Stewardship?

Antibiotic stewardship is a set of interventions put in place with the goal of reducing overall antibiotic use thereby combating antibiotic resistance. Antibiotic stewardship appears similar to environmental stewardship (Welchman, 1999) since a forward-oriented goal is kept in mind. However, antibiotic stewardship in the United States has primarily been focused on changing the use of antibiotics in institutions (i.e., inpatient antibiotic stewardship) and therefore has a more specific target than environmental stewardship (cf. Welchman, 1999). Practically, antibiotic stewardship requires additional microbiological testing and monitoring of the patient condition in order to assess whether antibiotics are needed, and if so, then what dose for what duration. For a physician conducting antibiotic stewardship, antibiotics are only appropriate if a patient's infection is confirmed via the microbiological testing and susceptibility testing that would confirm the efficacy of a selected antibiotic. Recognizing that antibiotic use is not always targeted to an infection, the activities of antibiotic stewardship also suggest that reducing overall antibiotic use would have a positive impact while not sacrificing a patient's health. Common activities in antibiotic stewardship programs include optimizing selection, dose, route of administration, and duration of antibiotics (Pakyz et al., 2014; Dyar et al., 2017). Typically, infectious disease physicians or specialized pharmacy staff will make calls to physicians giving recommendations designed to support good antibiotic stewardship policy.

Though antibiotic stewardship is a popular policy endorsed by the United States government and key infectious disease agencies, there are limits to the reach of antibiotic stewardship. At the level of the institution, antibiotic stewardship is just one of many interventions coming from various departments and divisions within the institution. Antibiotic stewardship programs vie for funding and support, building up an array of “champions” and “problem physicians” on either side of the cause. “Champions” are tasked with influencing the decisions (read: mindsets) of their colleagues in the direction of becoming stewards of antibiotics, meaning that they utilize antibiotics responsibly. “Problem physicians,” on the other hand, resist the advances of “champions” and continue prescribing antibiotics according to their own logics. While “problem physicians” are not seen as problematic by the institution as a whole, they are considered barriers to the implementation of antibiotic stewardship.

The heart of the social dynamics of antibiotic stewardship in the United States is the idea that “good behavior,” which here means responsible antibiotic use, can be achieved through careful, targeted attempts at changing the prescribing habits of other physicians. The focus is on bringing outlier physicians more in line with the prescribing habits of a department or division of the medical institution. For example, in the surgery department an individual is overprescribing cefazolin, giving two times the amount of antibiotic compared with their peers. This individual becomes a “problem physician” to the antibiotic stewardship program that is succeeding with the other physicians in the department but is not seeing change in this prescriber. According to the principles of behavioral economics and behavioral psychology, this individual can be brought into line with their peers through the utilization of “nudges” (Thaler and Sunstein, 2008) or small changes in the individual practices making up the institution.

### What Are Some of the Disciplinary Foundations of Antibiotic Stewardship?

Behind the structure of antibiotic stewardship lie tenets of behavioral psychology and behavioral economics best described in Thaler and Sunstein's *Nudge: Improving Decisions about Health, Wealth, and Happiness* (Thaler and Sunstein, 2008). Recently, behavioral economics has exploded on the scene as the intervention style of choice for nations and large institutions. Even before the popularity of behavioral economics, the underlying theories of behavioral psychology had a heavy influence on medical research (Pedwell, 2017: 14) leading to a focus on attitudes, perception, thoughts and behaviors. These epistemologies link up with the birth of behavioral economics, as Thaler describes in a recent Freakonomics appearance (Dubner, 2018) and as Scott Podolsky describes in relation to antibiotics in *The Antibiotic Era: Reform, Resistance, and the Pursuit of a Rational Therapeutics* (Podolsky, 2015). Thus, the antibiotic stewardship that we see promoted nationally appears to be continuing in the tradition of leaning heavily on the individualism that is prominent in both psychology and economics.

The disciplinary foundations of antibiotic stewardship have a heavy influence on the day to day practices of antibiotic stewards. The relationship between behavioral theories and antibiotic stewardship policy is exemplified in the tangible efforts at changing individuals, and thereby expecting to change overall trends in behavior. Thaler and Sunstein (2008) introduce Carolyn as an example. Carolyn learns that if she prominently displays healthier food in her school cafeteria, students tend to go for the healthier options. This example is a good representation of the logics behind behavioral economics which have become palpable among antibiotic stewardship researchers. Nudging students toward healthier lunch options is said to “…make ameliorative contributions to much bigger issues, from childhood obesity to adult heart health” (Pedwell, 2017).

Similarly, nudging prescribers toward better antibiotic choices can contribute to the much bigger issue of antibiotic resistance. Nudging promises low-cost, high-impact solutions. However, as Pedwell argues, citing Carolyn's cafeteria solution, “…such techniques do nothing to acknowledge the interrelated psychic, social, and economic factors that may play into cafeteria behavior and eating habits…from poverty, to academic pressure, to abuse and trauma, to sexism” (2017: 17). Importantly, these interrelated factors do not simply constitute context but influence understandings and behaviors of the individuals involved. In the following section, I introduce my fieldsite and describe the understandings of antibiotic stewardship leaders and antibiotic prescribing physicians at this site. By illuminating the social dynamics of antibiotic practice, I will show how antibiotic stewardship targeted at individual behavior is a reductionist approach that does not do justice to the real contexts of antibiotic use, leaving corresponding antibiotic stewardship interventions prone to failure.

## Methods

The research for this paper was conducted over an 18 months period at two adjacent medical institutions in the United States with outside support from the Wenner-Gren Foundation for Anthropological Research. The objective of the research was to understand antibiotic prescribing among hospital-based physicians. This objective was achieved by conducting participant observation and semi-structured interviews with specialists in infectious diseases (infectious disease practitioners and antibiotic stewards) and antibiotic prescribing non-specialists (intensive care unit practitioners). Ethnographic methods were chosen to illuminate the social milieu of the hospital through close observation and careful attention to cultural norms. Over 520 h of participant observation and over 39 h of semi-structured interviews were completed between July 2017, and December 2018.

### Setting

Two adjacent medical institutions in an urban midwestern city in the United States were chosen for this research. The first is a public teaching hospital with an over 20 years history of antibiotic control and antibiotic stewardship programs. The public teaching hospital shared an infectious disease fellowship program with the second institution, a private academic medical center with a more recent entrée into the world of antibiotic stewardship. The private academic medical center is a nationally-recognized center for orthopedic and geriatric care. Together, these institutions comprise a center for antibiotic stewardship as designated by the Centers for Disease Control and Prevention. This study is based at a single site for the purposes of analyzing specific iterations of antibiotic stewardship at a well-known duo of institutions, thus limiting the breadth of the study while allowing for a more in-depth look at local practice.

### Data Collection

As the researcher leading this study, I contacted infectious disease practitioners directly according to patient rounding schedules for the infectious disease consult service in the months of July 2017—January 2018. Once practitioners provided verbal consent, I joined individual infectious disease consult teams for their 2 weeks patient service. In total eight services were observed. Following the initial period of participant observation, I interviewed 25 infectious disease practitioners (attending physicians, fellows, and pharmacists). Though some interviewed participants were also observed in the first part of the study, select additional participants were added based on their research interests and involvement in antibiotic stewardship.

For the intensive care unit practitioners, similar methods were utilized. I contacted surgical intensive care unit practitioners directly according to patient rounding schedules for 1 week services in the months of April 2018—June 2018. Once practitioners provided verbal consent, I joined individual surgical intensive care unit consult teams for their 1 week patient services. In total 10 services were observed. At the end of each 1 week service I arranged to conduct a semi-structured interview with the attending practitioner (surgeon or anesthesiologist) and their physician fellow. The schedules for physician fellows follow a 1 month rotation, therefore fewer physician fellows were included in the study compared with attending practitioners.

### Data Analysis

For all periods of data collection, fieldnotes were taken during participant observation. At the end of every day, fieldnotes were typed into a document held within a qualitative analysis software (MAXQDA). Semi-structured interviews were recorded and transcribed at a later date. Thematic coding (Gibbs, 2007) of typed fieldnotes and semi-structured interviews was assisted by MAXQDA. First, a review was conducted through open coding of the typed data. Key themes that arose during open coding were solidified as overarching concept-driven codes that were then applied to the data. This allowed me to analyze the data inductively and perform a check on the initial open coding analysis. The data analysis was conducted onsite and as such I had contact with participants throughout the data analysis period (cf. Liberati et al., 2019). Participant feedback was solicited regarding the key themes and codes that were formed during data analysis. In order to protect participant anonymity, all data has been deidentified and pseudonyms are used throughout my written publications. The presentation of data in this article follows a thematic narrative approach (Emerson et al., 2011) aimed at ethnographic storytelling that leads to a culmination of central ideas in the text. As such, in the following you will find two ethnographic case studies followed by a discussion and analysis. The selected ethnographic case studies are representative in that they demonstrate key themes identified during the data analysis phase of research. Additionally, though the ethnographic case studies describe scenes from different medical institutions^2^ they are indicative of broader social dynamics and underlying beliefs found in many medical institutions (e.g., Charani et al., 2019).

### Ethical Issues

Ethical approval for the study was received prior to the start of data collection. All participants were informed about the research. All participants including the practitioners quoted in this paper provided verbal consent prior to involvement in the study and were informed that by giving their consent they may be included in a future publication of the study results. Furthermore, all participants were informed that they were free to leave the study at any point including during a scheduled interview. Participants mentioned in this article have been given pseudonyms used throughout the text as a safeguard to protect anonymity. The names of the medical institutions involved in this study are not disclosed to further protect the anonymity of participants.

## Limitations

This ethnographic research focused on two medical institutions in the same urban area of the United States. Therefore, suburban or rural medical institutions were not included in the data collection. Additionally, antibiotic use varies regionally and this research was conducted in a single region of the United States. Though this study did not aim to include these variables in the data collection, future research is needed to address how social dynamics are altered based on geographic region and type of medical institution.

## Ethnographic Case Study: Encountering Local Antibiotic Stewards

The following ethnographic case study shows that antibiotic stewardship is predicated on the notion that improving antibiotic use necessitates altering the mindsets, thoughts, and behaviors of individual antibiotic prescribers. The former Chair of the Division of Infectious Diseases had increased the visibility of antibiotic control programs, and later antibiotic stewardship, in the institution. An early committee on the subject had made a simple antibiotic switch that saved the hospital system millions. Successes such as these gave antibiotic stewardship notoriety amongst hospital system heads. The current chair, Dr. Martin, had been at his post for the past 5 years, and was attempting to increase the efficacy of antibiotic stewardship by fostering collaborative relationships with “champions” in diverse specialties. I met regularly with Dr. Martin in the early days of my ethnographic fieldwork. He explained, “For your champions, they have to be willing to say “This is not right.” They get to influence their colleagues, their patients, all the people they work with. They get to really champion that (good antibiotic use).” For example, the infectious disease pharmacist who often made calls for antibiotic stewardship purposes was improving the chain of communication to floor pharmacists who managed certain areas of the hospital (e.g., the medical intensive care unit pharmacist). Infectious diseases fellows were also involved in antibiotic stewardship, doing daily reviews of not only their own patient lists but antibiotic stewardship-specific lists. One such list involved all new cases of bacteremia (the presence of bacteria in the bloodstream) in the institution, which infectious disease fellows reviewed for antibiotic appropriateness and the potential need for an infectious disease consultation request. These additional pharmacists and fellows, through their involvement in antibiotic oversight, were recognized as antibiotic stewardship “champions.”

The general approach to antibiotic stewardship on a practical level was to find errors in the data (i.e., evidence of overuse or misuse in patient charts) and correct the error by speaking personally or through a formal channel such as consultation with the individual who had committed the error. I found it was a search and change mission that focused its efforts at ground level or the individual behaviors and habits of physicians. Throughout the course of my fieldwork, when trends became obvious to those on the antibiotic stewardship team, some additional steps might be taken such as giving a morning lecture to the targeted specialty or having a one-on-one conversation with the head of the targeted specialty. Additional methods of correction involve monitoring and restricting which antibiotics are available for use in the institution. By controlling the menu of antibiotic options for physicians, the stage is already set for a decision concordant with antibiotic stewardship policy. In other words, the behind-the-scenes work done by antibiotic stewards like Dr. Martin impacted the menu of options for physicians attempting to prescribe antibiotics.

When antibiotic stewardship approaches still fails to create change among other physicians, the individual physician mindsets and thought processes are thought to be at fault. An antibiotic steward described the limits to her involvement in a patient case:

“In the outpatient setting, you come in with a cold, you come in with a runny nose and a sore throat and a cough and you want antibiotics? There's no benefit. No! I'm not doing it. But in the inpatient setting it's not that cut and dried. It's not a “Yes” or “No.” There's so much gray. I've heard physicians say, “But is there a possibility that they would do better on the antibiotic? Because if there's even the slightest possibility then we'll give it.” It frustrates me because we don't live in a world of zero risk, you know, everything has a possible benefit and a possible risk. So we as individuals need to determine how comfortable we are with those possibilities.”

Here, again, the individual physician is seen as the deciding factor for antibiotic use. Antibiotic stewards in my research emphasize good decision making among individual prescribing physicians as an ultimate goal of their practice. For example, Dr. Martin invited me to observe a talk he gave to the obstetrics and gynecology group of the institution in late July. Here, he talked about having the capability of deciding for oneself when to switch antibiotics. “You have to decide, because it is your practice”^3^ he told them. Dr. Martin was walking the line between pushing for more appropriate use of antibiotics, a bread and butter antibiotic stewardship standard, and cornering the department into taking more responsibility for their antibiotic decisions without intervention from the antibiotic stewardship team. The combination of wanting physicians to choose antibiotics well and wanting to control the choices of physicians left Dr. Martin, and others involved in antibiotic stewardship, frustrated. During my observations this frustration often came to a head when discussing next steps for the antibiotic stewardship program.

In fact, Dr. Martin often approached conversations about antibiotic stewardship from the perspective displayed above. “What's wrong with us?” Dr. Martin asked me 1 day before an antibiotic stewardship meeting as he gestured toward the other individuals in the room. He continued in this vein. “We want to learn about how we can improve our (antibiotic stewardship) program…because I do think it is all in here (he points at his head).” It was not uncommon at my fieldsite for physicians to question their own behavior, though certainly it was more common to question the behavior of others they interact with. However, what Dr. Martin identifies as the error in this case is the behavior or himself and others as individuals. He assumes that the error is behavior affected by the mind in a negative way. The something wrong, here, is guiding individuals in the wrong direction, away from appropriate antibiotic use. According to this framework, altering the mindsets of individuals would thereby create change at the level of antibiotic prescribing. “What I want to do,” Dr. Martin says, “Is find out what it is that makes a physician behave the way they do. Mindsets, concepts, whatever it is so that we (the antibiotic stewardship team) can intervene and improve (antibiotic) stewardship.” The day Dr. Martin lectured to obstetricians and gynecologists in late July, he was intervening where he saw the error occurring, which was at the individual level. In the next section, I will show how the focus on individuals and individual behavior eschews understandings of antibiotic prescribing practice as a collective practice occurring within weighted institutional contexts.

## Ethnographic Case Study: Bucking Assumptions in the Surgical Intensive Care Unit

This ethnographic case study demonstrates how antibiotic decision-making in the surgical intensive care unit is carried out across multiple individuals and teams, thereby illuminating the limitations of a framing of antibiotic stewardship that focuses on individual behaviors. Surgeons at my fieldsite, as they are in much of the world, were known for their stubborn nature. Throughout my fieldwork, each medical institution had particular surgeons known to all rotating physicians. Certain names kept coming up in my notes. One recurring figure was Dr. Kline. The physician I was observing 1 week said, “Dr. Kline keeps *whipples* (patients who have undergone a Whipple procedure) 7 days so let's make sure we do that.” Another day in the unit, a different physician commented, “Dr. Kline is particular about pain control—ask him what he wants.” My notes continually referred to Dr. Kline as a surgeon with peculiar preferences for his patients, a “problem physician” who ensures their preferences are enacted.

One morning I was part of the group of resident physicians rounding with Dr. Tuttle on the surgical intensive care unit. These patients had undergone surgery and were not yet stable enough to begin recovery on the hospital floors or at home. Dr. Tuttle's team oversees the care of these patients and collaborates with the operating surgeons to decide trajectories for each patient's care. As we walk the floors that morning, I notice the following interaction unfold: Dr. Tuttle is engaged in conversation with the resident physicians as they discuss Mrs. Rodriguez, the current patient. Dr. Tuttle asks about fluids, chastising the resident physicians for not being more vigilant. “Fluids are like a vital in the intensive care unit, you need to trend those.” The new bacterial cultures were in, the resident physician copies down the results from the computer-on-wheels and conveys the message to Dr. Tuttle. The cultures showed growth, and the patient had a fever and hypotension. Now, vancomycin, cefepime, metronidazole,^4^ and micafungin could be peeled back to a streamlined course of antibiotics targeted to the culture results. The team discussed options, and Dr. Tuttle noted, “Dr. Kline always does 7 days.” The resident physician took note but nothing was decided at that moment.

We continued rounding on patients for another hour and a half. Later in the morning, as rounds died down, Sarah the team pharmacist checked in with the resident physicians. She clarified the dose and duration of antibiotics for several patients, including Mrs. Rodriguez. For Mrs. Rodriguez, Sarah left two options. If Dr. Kline does want to change the antibiotics, it'll be to this combination and this duration (Sarah put a paper down in front of the resident). If Dr. Kline doesn't want to change the antibiotics, go ahead and get rid of the micafungin. Either way, she instructed the resident physician, “Check in with me before you enter the changes.”

Dr. Tuttle and Sarah broke off to go to meetings while the resident physicians headed back to the work room to write their notes. I would often stay and write notes alongside the residents. After an hour of working independently, the resident physician in charge of Mrs. Rodriguez's case, Steve, picked up the work room phone and called one of Dr. Kline's resident physicians. Steve brought up the new bacterial culture results, Dr. Kline's resident physician confirmed that they've seen them. But there was a holdup. Unfortunately, Dr. Kline was in surgery at that moment. The choice was to try to reach Dr. Kline or move forward without his input. Dr. Kline's resident physician didn't ask to wait until Dr. Kline was out of surgery. Instead, with the bacterial culture results at hand Dr. Kline's resident physician decided that getting rid of micafungin was a good plan. There was no discussion of duration. Steve nodded, then confirmed the other antibiotics: vancomycin, cefepime, and metronidazole. Already, Dr. Tuttle's team was not solely responsible for the prescription of antibiotics for Mrs. Rodriguez. Steve, through his conversation with Dr. Kline's resident, had ensured that additional teams were involved.

The resident physicians continued working for another hour, then we broke for lunch. I returned to the work room at 1 p.m. to a new update in Mrs. Rodriguez's case. The infectious diseases consulting team on her case had put their notes in the medical record. This team commented on bacterial cultures, antibiotic selection, and antibiotic duration, among other specialty-related topics. The infectious diseases recommendations suggested that removing micafungin is indeed the first step for Mrs. Rodriguez. However, the infectious diseases consult team also recommended removing metronidazole on the basis that the bacterial culture results did not show evidence of microbes that would be targeted with metronidazole, thus rendering it useless in Mrs. Rodriguez's case. Finally, the infectious disease consult team suggested only two additional days of antibiotic, arguing that the patient had already received 3 days and a total of 5 days was all that was necessary per the institution recommendations. Now, even more input had been solicited for Mrs. Rodriguez's case. The expanding number of individuals involved in the decision-making had reached well-beyond the original prescribing physician.

At this point, Steve had gathered information from various teams regarding Mrs. Rodriguez's antibiotics. He wrote up his note in a hurry, he was being called in to another patient's room. The note was entered into the medical record, though the recommendations in his note were not put into action. Further action was required. Dr. Tuttle settled the case by signing the patient note and recommendations at just after 5 p.m. It was one of the last notes that she signed for the day. The completed recommendation? Dr. Tuttle commented that she agreed with Steve's characterization of the case and updates for the day. The antibiotics that will continue into tomorrow include vancomycin and cefepime, but not metronidazole or micafungin. Steve had written that the duration of antibiotics would total 7 days, or four additional days from the current date. Dr. Tuttle signed off on this duration. In the beginning, we have one team, even one physician, suggesting the course of action for a patient. Often, initial reactions like the one from Dr. Tuttle are taken as the most significant behavior in antibiotic decision-making. However, as I demonstrate here, this initial reaction was not the final say. In fact, the number of individuals involved expanded to include multiple teams. Later, the number of individuals shrunk again until the final action in the case was made: Dr. Tuttle signed the antibiotic recommendations.

## Discussion

In “Encountering Local Antibiotic Stewards,” Dr. Martin searches for the reasons behind individual physician “behaviors” and “habits.” With his research agenda, Dr. Martin is determined to identify and ultimately change individual prescribers to come more in line with antibiotic stewardship recommendations. With the obstetrics and gynecology group, Dr. Martin attempts to reinforce the physicians' power and responsibility to prescribe appropriately, telling them “…it's your practice.” He walks through case studies of obstetrics and gynecology patients where antibiotics were prescribed and poses questions to the practice group about what antibiotic they would choose. By retraining the physicians, Dr. Martin is addressing what he sees to be the underlying concern with antibiotic overuse and misuse: there is something wrong with us.

Dr. Martin is ultimately interested in improving antibiotic stewardship at the medical institution where he works. Both segments of the literature (ex. Meeker et al., 2014) and his own personal instincts tell him that it is individuals that need to be changed. Thus, in my conversations with Dr. Martin, the focus continually returns to the individual physician and more specifically, their thought processes. The antibiotic stewardship goals that Dr. Martin creates while I am at my fieldsite follow the precipitated notions regarding who is at fault with antibiotic overuse and misuse (i.e., “bad” prescribers) and how they can be changed into good prescribers (i.e., “champions”).

Antibiotic stewardship based on Dr. Martin's question “What's wrong with us?” involves targeting individuals from every facet of the institution. It can be considered a holistic approach in one sense, that individuals from every specialty and hospital floor are targeted. However, it is ultimately a reductionist approach since it rarely addresses the collaborations and interactions shaping antibiotic use in medical institutions. Though many antibiotic stewardship programs operate as though the social and institutional dynamics at play simply constitute context, in fact these interrelated factors contour the processes of how antibiotics are prescribed on a daily basis.

In “Bucking Assumptions in the Surgical Intensive Care Unit,” we find a scene in which multiple physicians at various points in time and at different locations within the medical institution are involved in making antibiotic decisions for the surgery patient Mrs. Rodriguez. There are three key elements demonstrating the collective nature of antibiotic decision making in Mrs. Rodriguez's case. First, Dr. Kline isn't really involved in making antibiotic decisions. We see that he is unavailable during the call between teams asking about antibiotic preferences. Further, we do not see any later intervention on Dr. Kline's behalf to change what others have prescribed to the patient. Thus, Dr. Kline has effectively delegated responsibility to the resident physician. The resident physician from Dr. Kline's team, though he could have waited to confirm with Dr. Kline which antibiotic and what duration of antibiotic were needed, took the initiative to make recommendations himself. This scenario can be compared to Charani et al. (2019), where antibiotic decisions for surgical patients are similarly delegated to junior physicians.

Second, other physicians and pharmacists are involved in making decisions regarding Mrs. Rodriguez's antibiotics. Though Dr. Kline does not have an active role, Steve, Dr. Tuttle, and Sarah all have significant influence over the decision to give Mrs. Rodriguez vancomycin and cefepime for a total of 7 days. In the morning during patient rounds, these individuals were communicating and making suggestions for how to reach a decision on the antibiotic course. Steve took notes on what was being said by Dr. Tuttle and the pharmacist Sarah. Later in the day Steve had written out his note including the antibiotic orders. Dr. Tuttle then signed off on this note and it became a signed order in the electronic medical record, meaning that the antibiotic was scheduled to be given as ordered^5^. The engagement of multiple individuals in the case demonstrates the collective nature of antibiotic decision making. These social determinants are important considerations that do not fit easily into current formulations of antibiotic stewardship.

A third key element in “Bucking Assumptions in the Surgical Intensive Care Unit” is how time pressures and the structure of medical practice impact the antibiotic decision. Importantly, had any of these individuals been called away or with another service that day (ex. Sarah often rotates which intensive care unit team she works with), the outcome could have been different. Additionally, had the team had several days to ruminate over the antibiotic choices, the outcome could have been different. During my research, I was constantly aware of the time pressures that physicians were placed under. Since notes in the medical record must be signed within the time limit set by insurance companies, notes end up getting signed by end of day (or night). Thus, the requirements of the structure of medical practice also shape the manner in which antibiotic decisions are made.

In the context of the tendency of antibiotic stewards to locate the power of decision making within individual physicians, and in light of evidence to the contrary, a deeper analysis of the data reveals complex social dynamics and institutional structures of practice that are otherwise invisible. There has been emphasis placed on the “behaviors” and “habits” of antibiotic prescribers that hinder antibiotic stewardship such as “defensive prescribing” (Mol et al., 2006) and “stealth dosing” (La Rosa et al., 2007). Furthermore, and for a variety of reasons including the social dynamics of medical practice, some physicians actively avoid following antibiotic stewardship recommendations, performing “workarounds” (Szymczak et al., 2019). Antibiotic stewardship interventions to address misuse and overuse of antibiotics by physicians have largely targeted these “bad” individual physicians.

## Conclusion

The principles of behavioral economics suggest that by altering the conditions of the environment surrounding an individual one can influence that individual in the direction of a more favorable decision (Thaler and Sunstein, 2008). Meanwhile, social psychology encourages a more inward look at the rationales for behaviors and habits that individuals have (see Pedwell, 2017). Both of these approaches are inadequate to account for what really happens. Yet, antibiotic stewardship has a history of targeting individual physicians based on the underlying theoretical assumption that antibiotic decision making is an isolated act made in the mind of a physician. This study shows the fallacy of assuming antibiotic prescribing is an action completed by individuals by contrasting the common view represented by Dr. Martin with the ethnographic case study of the team working on Mrs. Rodriguez's antibiotic prescription.

Dr. Martin's perspective presented in this article is not unique among antibiotic stewards. In fact, it was the common view at my field site. In conclusion, I argue that while antibiotic stewardship programs often target individual physician prescribers, *antibiotic prescribing is a collective practice* influenced by social and material surroundings. Rather than just focus on the “behavior” and “habits” of physicians, the complex social dynamics present in the medical institution are actually more representative of where decisions regarding antibiotic use are made and signed off on. The ethnographic data illuminate (1) how entrenched the idea of individual prescribers is at my field site, and (2) how difficult it is to give credit to a single prescriber given the other individuals and institutional surroundings that direct decision making. Thus, while the antibiotic steward's understanding of the way to change physician prescribing behavior follows the tenets of behavioral economics and social psychology suggesting that problems are the result of individuals making bad choices, I have argued here using iterative and inductive research (cf. Karen O'Reilly, 2005) that deeper social dynamics in physician practice operate as agents shaping the conditions and determinants surrounding antibiotic use.

To optimize antibiotic use, antibiotic stewardship programs must appreciate the historic lack of input from the social sciences (particularly the qualitative social sciences, see Smith, 2015) that contributes to an underappreciation of the collective nature of antibiotic use (Chandler, 2019). Some antibiotic stewards have recognized that a one-size-fits-all program does not meet the needs of each culture and context (Jeffs et al., 2015; see “bespoke stewardship” Charani et al., 2019). I would like to suggest that beyond valuing context, antibiotic use can be optimized by reassessing where we consider to be the locus of antibiotic decision making (i.e., with the individual or the collective). We can begin to think of antibiotic prescribing as an activity occurring between persons amidst an institution harboring specific practices, physical spaces, and time pressures. For the antibiotic steward, this might mean changing the targets of antibiotic stewardship interventions. Furthermore, antibiotic stewardship programs could be pressed to reexamine existing notions of antibiotic prescribing processes by conducting observational and comparative research in their own local settings. Going forward, moving from the perception of antibiotic prescribing as something that is decided in our minds to something that unfolds and arises in context is critical.

## Data Availability Statement

The datasets generated for this study are available on request to the corresponding author.

## Ethics Statement

The studies involving human participants were reviewed and approved by The Institutional Review Board of John H. Stroger Jr. Hospital of Cook County. Written informed consent for participation was not required for this study in accordance with institutional requirements.

## Author Contributions

KR conducted the research for and wrote this article in its entirety.

### Conflict of Interest

The author declares that the research was conducted in the absence of any commercial or financial relationships that could be construed as a potential conflict of interest.

## Footnotes

^1^The Centers for Disease Control and Prevention now recommend that every medical institution have an antibiotic stewardship team comprised of individuals specially trained to monitor and advise on antibiotic use.

^2^The first ethnographic case study took place at the public teaching hospital while the second ethnographic case study is a composite case based on participant observation in the surgical intensive care unit at the private academic medical center. Though the institutions varied in their organizational structure and resources, several key practitioners were employed at both institutions and the antibiotic stewardship teams had a close working relationship. The foundational principles of antibiotic stewardship at both institutions were more similar than they were different.

^3^This approach is reminiscent of Former United States President Ronald Reagan's War on Drugs slogan: “Just say No.” The generally neoliberal assumption is that people are free to make choices and thus just need a slight push to make the right decision. Behavioral economics draws on some of these principles (McMahon, 2015).

^4^This antibiotic was referred to as Flagyl© at my fieldsite but I am keeping the references uniform as with the other antibiotics I have used generic names.

^5^Though an antibiotic order is placed in the electronic medical record, there are many steps that have to be taken for it to actually be administered by the nursing staff. How and when antibiotics are actually administered is not within the purview of this article as I have focused on how and when antibiotics are prescribed.
